# Harnessing Postbiotics to Boost Chemotherapy: N-Acetylcysteine and Tetrahydro β-Carboline Carboxylic Acid as Potentiators in Pancreatic and Colorectal Cancer

**DOI:** 10.3390/cancers18030369

**Published:** 2026-01-25

**Authors:** Vanessa Rodriguez, Annacandida Villani, Margarida Sénica, Concetta Panebianco, Valerio Pazienza, Ana Preto

**Affiliations:** 1 Centre of Molecular and Environmental Biology (CBMA), Department of Biology, University of Minho, Campus of Gualtar, 4710-057 Braga, Portugal; pg49131@alunos.uminho.pt (V.R.); pg54390@alunos.uminho.pt (M.S.); 2 IBS—Institute of Science and Innovation for Bio-Sustainability, University of Minho, Campus of Gualtar, 4710-057 Braga, Portugal; 3 Division of Gastroenterology, Fondazione IRCCS Casa Sollievo della Sofferenza, 71013 San Giovanni Rotondo, Italy; villaniannacandida@yahoo.it (A.V.); panebianco.c@gmail.com (C.P.)

**Keywords:** colorectal cancer, pancreatic cancer, N-acetylcysteine, tetrahydro β-carboline carboxylic acid, postbiotics

## Abstract

Background: Pancreatic cancer (PC) and colorectal cancer (CRC) are among the most lethal malignancies, with growing evidence pointing to the gut microbiota’s role in their progression. This study aimed to explore the anticancer potential of two microbiota-derived postbiotics, N-acetylcysteine (NAC) and tetrahydro β-carboline carboxylic acid (THC), in targeting some hallmark traits of PC and CRC, both as standalone agents and in combination with standard chemotherapeutics (gemcitabine for PC and 5-fluorouracil (5-FU) for CRC). Methods: Cell viability assays and IC_50_ determination was assessed using either the Muse™ Count & Viability Kit or the Sulforhodamine B assay; cell death was determined by Annexin V/Propidium Iodide and cell cycle assessed by Propidium Iodide was analyzed by flow cytometry. Results: Here, we found that NAC selectively reduced the viability of PC cells BxPC-3 without triggering apoptosis, while effectively inducing apoptosis in PC cells Panc-1 and in CRC cell lines. THC exhibited stronger anticancer activity, inhibiting proliferation and promoting apoptosis in all tested PC and CRC cells, even at lower concentrations. Combination treatments yielded promising enhancement effects. NAC enhanced the cytotoxicity of gemcitabine in Panc-1 cells through increased apoptosis. NAC, when combined with 5-FU, also increased apoptosis of CRC cells. THC further potentiated gemcitabine’s impact on Panc-1 cells by increasing apoptosis and by inducing cell cycle changes in BxPC-3. In the CRC model, THC co-treatment with 5-FU reduced cell viability and increased apoptosis in all cells. Conclusions: These findings provide preliminary in vitro evidence supporting the potential of integrating microbiota-derived postbiotics with conventional chemotherapy both in PC and CRC.

## Simple Summary

Chemotherapy is widely used to treat cancer, but its effectiveness can be limited by drug resistance and side effects. In recent years, compounds produced by our gut bacteria, also known as postbiotics, have gained attention for their potential to support cancer treatment. In this study, we investigated whether two postbiotics, N-acetylcysteine and tetrahydro β-carboline carboxylic acid, could influence the growth of pancreatic and colorectal cancer cells. We tested their effects on cell survival, cell cycle regulation, and programmed cell death, both alone and in combination with common chemotherapy drugs. Our results showed that both postbiotics reduced cancer cell growth, with tetrahydro β-carboline carboxylic acid showing particularly strong effects. When combined with chemotherapy, both postbiotics enhanced the anticancer activity of the drugs in certain cell types. These findings provide preliminary in vitro evidence suggesting that postbiotics may serve as potential adjuvants to chemotherapy.

## 1. Introduction

Cancer remains the second leading cause of death globally, and despite advances in treatment, conventional therapies such as chemotherapy are often limited by severe side effects and the frequent emergence of drug resistance [1]. These limitations underscore the urgent need for more effective and tolerable therapeutic strategies.

Pancreatic cancer (PC) and colorectal cancer (CRC) are among the leading causes of morbidity and mortality worldwide. In 2022, PC was the sixth leading cause of cancer-related death and the fourteenth most common type of cancer worldwide, with an estimated 510,922 new cases and 467,409 deaths all over the world [2]. In the same year, CRC ranked as the second leading cause of death and the third most commonly diagnosed cancer, accounting over 1.9 million of new cases and 904,000 deaths globally [2]. Both PC and CRC develop through the accumulation of genetic alterations. In PC, the most frequent affected genes include Kirsten rat sarcoma viral oncogene homolog (*KRAS*) (95%), Cyclin-dependent kinase inhibitor 2A (*CDKN2A*) (50%), Tumor protein 53 (*TP53*) (50–75%), and Mothers against decapentaplegic 4 *SMAD4* (50%) [3,4,5], while CRC is influenced by genetic alterations in B-Raf proto-oncogene (*BRAF*) (8–15%), Phosphatidylinositol-4,5-bisphosphate 3-kinase catalytic subunit alpha (*PI3KCA*) (15–20%), *KRAS* (40%), and Neuroblastoma RAS viral oncogene homolog (*NRAS*) (1–3%) [6,7,8]. Standard treatment strategies for the treatment of these two types of cancer involve surgical resection followed by chemotherapy with gemcitabine for PC [9] and 5-fluorouracil (5-FU) for CRC [10]. However, both treatments are limited due to severe side effects and drug resistance [9,11,12].

Recent research has highlighted gut microbiota as a novel and influential player in cancer development and therapy response. Beyond its traditional roles in immune system modulation, xenobiotic metabolism, and toxin elimination, microbiota has emerged as a key factor in tumor suppression and progression. Gut microbiota influences cancer progression through multiple mechanisms, such as the modulation of systemic and local immunity, the production of metabolites affecting cancer cell signaling, the metabolism of xenobiotics like chemotherapy drugs, and the maintenance of intestinal barrier integrity [13,14,15]. Disruptions in microbial composition, referred to as dysbiosis, have been associated with several malignancies, including PC and CRC [16]. Moreover, the emerging field of pharmacomicrobiomics reveals a bidirectional relationship between microbiota and cancer therapies; while certain microbial profiles can enhance the efficacy of anticancer treatments, these treatments can in turn alter microbiota, often worsening dysbiosis [17,18]. This bidirectional relationship creates opportunities for microbiota-targeted interventions to optimize cancer therapy outcomes.

Given this interplay, restoring a favorable microbiota has become a promising adjunct strategy in cancer care. Approaches such as probiotics, prebiotics, and postbiotics are being explored to modulate the gut ecosystem. Probiotics, live microorganisms that confer health benefits, have shown potential in reducing chemotherapy-induced toxicity, enhancing therapeutic efficacy, and even preventing tumor development. For instance, in a murine model of pancreatic ductal adenocarcinoma (PDAC) treated with gemcitabine, gut dysbiosis was observed alongside an increase in pro-inflammatory bacteria [18]. However, the administration of a probiotic blend (PRO2101) in a follow-up study mitigated these effects, improving microbiota composition and enhancing therapeutic outcomes [19].

Further investigation into the individual strains within the probiotic blend (PRO2101), particularly *Bifidobacterium bifidum* and *Bifidobacterium breve*, revealed that their cell-free supernatants (CFSs) could prevent epithelial–mesenchymal transition (EMT) in BxPC-3 pancreatic cancer cells, especially when combined with gemcitabine. This led to metabolomic profiling of the CFSs to identify specific metabolites, or postbiotics, that might be responsible for the observed anticancer effects [19]. N-acetylcysteine (NAC) and tetrahydro β-carboline carboxylic acid (THC) were specifically selected based on their relative abundance in the metabolic profiles of the most effective probiotic strains, *B. bifidum* and *B. breve*, and the existing literature documenting their potential anticancer properties [20,21].

Postbiotics, defined as non-viable microbial products or metabolic byproducts that confer health benefits, offer several practical advantages over probiotics, including greater stability, easier storage, and faster biological activity [18,19]. This study investigates the anticancer potential of the postbiotics NAC and THC in PC and CRC cell models harboring different genetic backgrounds. Cell line selection was strategically designed to represent key genetic drivers in PC and CRC. In the case of PC, we selected BxPC-3 (*KRAS*^WT^) and Panc-1 (*KRAS*^G12D^) since *KRAS* mutations are constitutively active in approximately 95% of pancreatic cancer [7,22,23]. For CRC, we included SW480 (*KRAS*^G12V^) and RKO (*BRAF*^V600E^), which represent two of the most common mutually exclusive mutations in CRC with *KRAS* mutations appearing in 40% of cases and *BRAF* mutations appearing in 8–15% of patients [7,22,23]. These oncogenic mutations activate downstream signaling pathways, most notably, phosphatidylinositol 3-kinase (PI3K) and mitogen-activated protein kinase (MAPK), which regulate cell proliferation, differentiation, and survival [24,25,26,27,28,29,30,31]. This selection strategy allows assessment of whether postbiotic responses differ based on oncogenic driver mutations and cellular phenotypes.

We specifically aimed to investigate the individual effects of NAC and THC on cancer cell viability and some cancer hallmark behaviors, as well as their ability to enhance the efficacy of standard chemotherapeutic agents, gemcitabine for PC and 5-FU for CRC.

## 2. Materials and Methods

Compounds analyzed. N-acetyl-L-cysteine (Sigma-Aldrich, St. Louis, MO, USA) was dissolved in sterile water. The compound 1,2,3,4-Tetrahydro β-carboline-1-carboxylic-acid (TRC, North York, ON, USA) was dissolved in 100% dimethyl sulfoxide (DMSO) which was purchased from Honeywell (Charlotte, NC, USA). The 5-fluorouracil (5-FU) chemotherapeutic agent,(Sigma-Aldrich, St. Louis, MO, USA) was dissolved in DMSO; the chemotherapy drug gemcitabine(Sigma-Aldrich, St. Louis, MO, USA) was dissolved in sterile water.

Cell lines and conditions. The experiments were carried out in two human-derived CRC cell lines—RKO (*BRAF*^V600E^ mutation; valine at position 600 is mutated to glutamic acid in the *BRAF*^V600E^ mutant) and SW480 (*KRAS*^G12V^ mutation; glycine at position 12 is mutated to valine in the *KRAS*^G12V^ mutant)—a normal colon epithelial cell line derived from the human colon, NCM460, two human-derived PC cell lines: BxPC-3 (*KRAS*^WT^) and Panc-1 (*KRAS*^G12D^ mutation; glycine at position 12 is mutated to aspartate in the *KRAS*^G12D^), and an hTERT-immortalized fibroblast cell, BJ-5ta. All PC and CRC cell lines and BJ-5ta were purchased from American Type Culture Collection (Manassas, VI, USA) and the NCM460 cell line was purchased from INCELL’s (San Antonio, TX, USA).

Cell lines were maintained in either Petri dishes (100 × 20 mm) or in T25 cm^2^ polystyrene tissue culture flasks with Roswell Park Memorial Institute (RPMI) 1640 (Biowest, Nuaillé, France) for SW480, NCM460, and BxPC-3, and Dulbecco’s Modified Eagle’s Medium (DMEM) (Biowest, Nuaillé, France) for RKO, Panc-1, and BJ-5ta. Both culture media were supplemented with 10% (*v*/*v*) fetal bovine serum (FBS) (Capricorn Scientific, Ebsdorfergrung, Germany) and 1% (*v*/*v*) of penicillin and streptomycin (PenStrep), an antibiotic solution (Biowest, Nuaillé, France). In the case of the BJ-5ta cell line, DMEM was supplemented with 10% (*v*/*v*) of FBS, one part of 199 Medium (PAN Biotech, Aidenbach, Germany), and 100 µL of hygromycin (Invitrogen, Waltham, MA, USA). For all the assays, all cell lines were grown to 70–90% confluence at a 37 °C, 5% CO_2_ incubator with humidified atmosphere. Three independent experiments were conducted in each cell line with each compound for all the assays. For 6-well plates, all cell lines were seeded at a density of 3.5 × 10^5^ cells/mL; meanwhile, for 24-wellplates, all cell lines were seeded at a density of 1 × 10^5^ cells/mL.

Cell viability assays and IC_50_ determination. To evaluate the effects of the postbiotics on the tested cell lines and to determine the half-maximum inhibitory concentration (IC_50_), cell viability was assessed using either the Muse™ Count & Viability Kit (Catalog No. MCH100102) (Cytek Biosciences, Fremont, CA, USA) or the Sulforhodamine B (SRB) assay. Two distinct assays were used because the experiments involving PC cell lines were conducted at “Fondazione Casa Sollievo della Sofferenza”, while studies performed on CRC cell lines and control cell lines were conducted at the Centre of Molecular and Environmental Biology. To evaluate cell growth and calculate the IC_50_, cells were seeded in 24-well plates and left to adhere for 24 h, to later incubate with different concentrations of the compounds for 48 h. The range of concentrations varied for each compound and cell line, the maximum concentration tested being 180 mM and the minimum being 0.5 mM. For each cell line and compound, a negative control was performed where the cells were exposed to complete growth medium with the vehicle in the highest concentration tested (H_2_O concentration in conditions with NAC and DMSO concentration in conditions with THC) and this was considered as 100% cell growth. After a 48 h treatment with different compounds, two approaches were used depending on the assay. For analyses performed with the Muse™ Cell Analyzer, the adherent cells were washed with 1× PBS, harvested using trypsin 0.05% (*v*/*v*) (Biowest, Nuaillé, France), resuspended in complete medium, and stained with the Count & Viability Reagent (200×) from the Muse™ Count & Viability Kit (Catalog No. MCH100102). This reagent differentially stains viable and non-viable cells based on membrane permeability to two DNA-binding dyes: a membrane-impermeant dye that stains only cells with compromised membrane integrity (dead or dying cells) and a membrane-permeant dye that stains all nucleated cells, allowing distinction between intact cells, debris, and non-nucleated particles. For the SRB assay, the Sulforhodamine B dye (Sigma-Aldrich, St. Louis, MO, USA), binds stoichiometrically to basic amino acid residues of cellular proteins. After fixation and staining, the bound dye is extracted under mild acidic conditions, and absorbance is measured at 540 nm in a Molecular Devices SpectraMax Plus 384 Microplate Reader (Molecular Devices, San Jose, CA, USA). The absorbance values are proportional to the total cellular protein content and thus reflect the number of viable cells [32]. Moreover, to calculate the selectivity index (SI) the IC_50_ values of each compound for normal cells, BJ-5ta and NCM460, were divided by the corresponding PC and CRC cell lines, respectively [33].

Enhancement of the effect of gemcitabine or 5-FU in cell proliferation. To observe if each compound enhanced the effect of the chemotherapy drug, gemcitabine in the two PC (BxPC-3 and Panc-1) and 5-FU in the two CRC (SW480 and RKO) cell lines, the Muse™ Cell Analyzer (Cytek Biosciences, Fremont, CA, USA) and SRB assay were performed, respectively. Similarly to the procedure performed to determine the IC_50_ of each compound, a 48 h treatment was performed in each cell line with each compound at their IC_50_ concentration alone and mixed with gemcitabine for the PC cell lines or with 5-FU for the CRC cell lines. The negative control was the same as the one used in the previous assay and for a positive control, the cells were treated with either gemcitabine or 5-FU for PC and CRC cell lines, respectively.

Cell death assessed by Annexin V/Propidium Iodide. When evaluating the induction of cell death, the cells were seeded in 6-well plates and after 24 h incubation the cells were treated, and left to incubate for another 48 h with the following: complete growth medium with the vehicle in the highest concentration tested (negative control), IC_50_ concentration of the compounds, chemotherapy agent (positive control), and these two latter conditions combined. Annexin V (AV) is a calcium-dependent phospholipid-binding protein with a high affinity for phosphatidylserine (PS); therefore, in the early stages of apoptosis, when the externalization of PS molecules occurs, AV binds to them, which helps measure its externalization (Invitrogen, Waltham, MA, USA). Moreover, a marker, such as propidium iodide (PI) (Sigma-Aldrich, St. Louis, MO, USA), was used to detect necrosis by measuring the structural integrity of the cell membrane. Therefore, this method differentiates normal/non-apoptotic cells from apoptotic cells, including early and late apoptotic cells. After 48 h, the cells were washed with 1x PBS, harvested using trypsin 0.05% (*v*/*v*), resuspended in complete medium, incubated with both dyes at RT in the dark, and analyzed by the Flow Cytometer (Beckman Coulter, Brea, CA, USA).

Cell cycle analysis by flow cytometry. Similarly, as performed in the previous assay, the same four treatments were performed in 6-well plates and left for 48 h in each cell line (SW480, RKO, BxPC-3, and Panc-1) with each compound. After 48 h treatment, the cells were washed with 1x PBS, collected using 0.05% trypsin, and resuspended in medium. The suspensions were centrifuged, the pellet was resuspended in 1x PBS, and the cells were fixed with ice-cold 96% (*v*/*v*) ethanol, which was added dropwise to the suspension while slowly vortexing and incubated at −20 °C. After the incubation, the samples were once again centrifuged and washed with 1x PBS. A final centrifugation was performed; the pellet was resuspended, treated with ribonuclease A (Sigma-Aldrich, St. Louis, MO, USA), and stained with PI.

Statistical analysis. Statistical analysis was performed with the GraphPad Prism version 8.4.3 for Windows (GraphPad Software, San Diego, CA, USA). A nonlinear regression analysis was performed for each compound in each cell line using a best-fit approach to determine the IC_50_ values. Data are expressed as mean ± standard deviation (SD) of the mean from at least three independent experiments. Student’s *t*-test was used to assess statistical differences among groups comparing individual treatments with combination treatments. Enhancement of combination effects was assessed through comparative statistical analysis between single and combination treatments. Statistical significance was assumed as *p*-values < 0.05 (*), *p*-values < 0.01 (**), or *p*-values < 0.001(***) for a confidence level of 95%.

## 3. Results

### 3.1. Evaluation of Cytotoxic Effects of NAC and THC on Pancreatic and Colorectal Cancer Cell Lines

To evaluate the anticancer potential of the postbiotics NAC and THC, dose–response studies were conducted across a panel of pancreatic (BxPC-3 and Panc-1) and colorectal (SW480 and RKO) cancer cell lines. Two non-cancerous cell lines, BJ-5ta (immortalized fibroblasts) and NCM460 (normal colon epithelial cells) served as the controls for PC and CRC cell lines, respectively.

**Figure 1:**
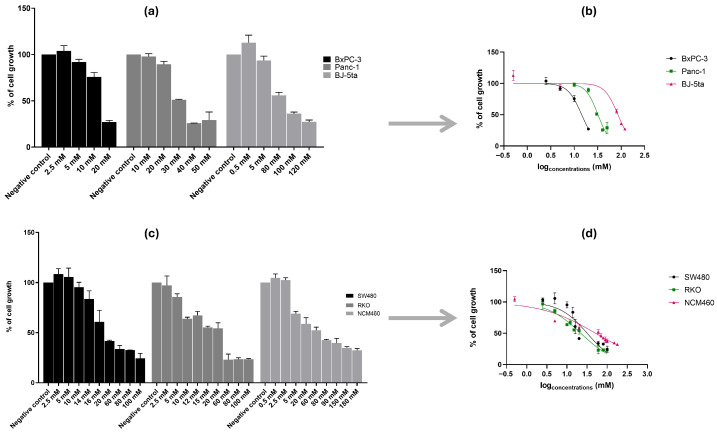
Effect of N-acetylcysteine (NAC) on cell growth in pancreatic cancer (PC) cell lines (BxPC-3 and Panc-1), colorectal cancer (CRC) cell lines (SW480 and RKO), and corresponding control cell lines (BJ-5ta and NCM460). Panels (**a**,**c**) show bar graphs of cell growth (%) ± S.D. across NAC concentrations, while panels (**b**,**d**) show nonlinear regression curves of cell growth (%) ± S.D. versus log [NAC]. Panels (**a**,**b**) correspond to PC lines and their control; panels (**c**,**d**) correspond to CRC cell lines and their control. NAC concentrations ranged from 0.5 to 120 mM (PC) and 0.5–180 mM (CRC). For PC cell lines, we used the Muse™ Count & Cell Viability Reagent and for CRC cell lines, we performed the Sulforhodamine B assay.

**Figure 2:**
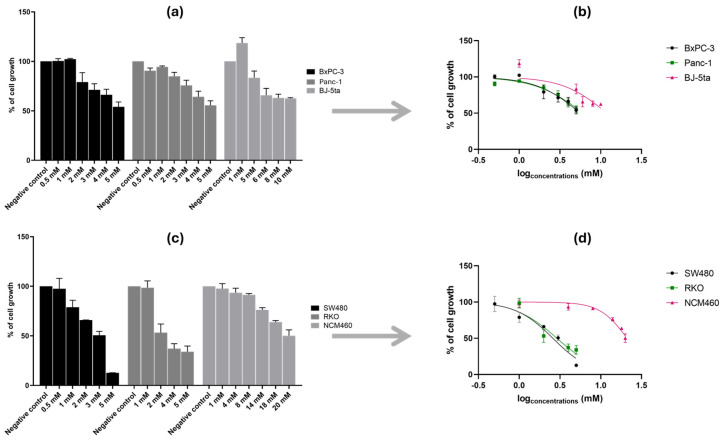
Effect of tetrahydro-β-carboline carboxylic acid (THC) on cell growth in pancreatic cancer (PC) cell lines (BxPC-3 and Panc-1), colorectal cancer (CRC) cell lines (SW480 and RKO), and corresponding control cell lines (BJ-5ta and NCM460). Panels (**a**,**c**) show bar graphs of cell growth (%) ± S.D. across THC concentrations, while panels (**b**,**d**) show nonlinear regression curves of cell growth (%) ± S.D. versus log [THC]. Panels (**a**,**b**) correspond to PC lines and their control; panels (**c**,**d**) correspond to CRC cell lines and their control. THC concentrations ranged from 0.5 to 10 mM (PC) and 0.5–20 mM (CRC). For PC cell lines, we used the Muse™ Count & Cell Viability Reagent, whilst for CRC cell lines, we performed the Sulforhodamine B assay.

Cell viability assays using increasing concentrations of NAC and THC (from a range of 0.5 to 180 mM) revealed a dose-dependent cytotoxic effect on all cancer cell lines tested. The IC_50_ was calculated for each cell line (Figure 1 and Figure 2; Table 1 and Table 2). The selectivity index (SI) was calculated to assess the compounds’ preferential cytotoxicity toward cancer cells over normal cells [33].

#### 3.1.1. N-Acetylcysteine Inhibits the Growth of Pancreatic and Colorectal Cancer Cell Lines

For PC cell lines, distinct concentration ranges were required: BxPC-3 cells responded to lower doses (2.5–20 mM), while Panc-1 cells required higher concentrations, up to 50 mM, to achieve a comparable effect (Figure 1a). The corresponding IC_50_ curves (Figure 1b) revealed greater sensitivity to NAC for BxPC-3 cells, as reflected by a lower IC_50_ value compared to Panc-1 (Table 1). The difference in sensitivity observed between these two cell lines might be caused by their distinct genetic and phenotypic characteristics, with BxPC-3 being *KRAS*^WT^ with epithelial features and Panc-1 harboring a *KRAS*^G12D^ mutation with more mesenchymal characteristics.

In CRC cell lines, a broader range (2.5–100 mM) was tested due to their reduced sensitivity to NAC (Figure 1c). These higher concentrations ranges were determined empirically during preliminary dose–concentration findings, as CRC cell lines exhibited lower sensitivity to NAC compared to PC cell lines, requiring higher concentrations to achieve sufficient cytotoxic effects and establish reliable IC_50_ values. This differential sensitivity may relate to intrinsic differences in cellular metabolism, basal oxidative stress levels, and genetic backgrounds between pancreatic and colorectal cancer models. As shown in Figure 1d, RKO cells were slightly more responsive to NAC than SW480, which is reflected in the modest differences in IC_50_ values (Table 1).

Control cell lines also exhibited a dose-dependent response but at notably higher NAC concentrations: BJ-5ta (0.5–120 mM) and NCM460 (0.5–180 mM) (Figure 1a,c). Accordingly, both controls showed higher IC_50_ values than their cancerous counterparts, resulting in SI values greater than one (Table 1). While this indicates that NAC preferentially reduced the viability of cancer cells while sparing normal cells, suggesting selectivity toward cancer cells, in vivo validation is necessary to confirm cancer selectivity. Based on these findings, further functional assays were performed to explore the effects of NAC on the growth of BxPC-3, Panc-1, SW480, and RKO cells.

#### 3.1.2. Tetrahydro β-Carboline Carboxylic Acid Influences the Cell Growth of Pancreatic and Colorectal Cancer Cell Lines

For THC, concentrations ranging from 0.5 to 20 mM were tested, and a clear dose-dependent reduction in cell growth was observed in both PC and CRC cell lines (Figure 2). For the cancer cell lines, concentrations between 0.5 and 5 mM were tested, while for the control cell lines, higher ranges were required: BJ-5ta was tested from 1 to 10 mM, and NCM460 from 1 to 20 mM (Figure 2a,c). These ranges were chosen to allow comparison of the dose-dependent effects between normal and cancer cells.

In PC cell lines, the IC_50_ curves (Figure 2b) showed that Panc-1 had a slightly higher IC_50_ value than BxPC-3 (Table 2), indicating that BxPC-3 was more sensitive to THC. A similar effect was observed in CRC cell lines, where both SW480 and RKO were inhibited to a similar extent; still, the IC_50_ curve of SW480 was slightly lower than that of RKO (Figure 2d), which corresponds to the lower IC_50_ value of SW480 shown in Table 2.

When comparing the two compounds, THC showed lower IC_50_ values than NAC across all six cell lines (BxPC-3, Panc-1, BJ-5ta, SW480, RKO, and NCM460) (Table 1 and Table 2). In both control cell lines, BJ-5ta and NCM460, the IC_50_ values were higher than those of their corresponding cancer cell lines, resulting in SI values greater than one (Table 2). Notably, for CRC cell lines, the SI values were higher with THC than with NAC, while in PC cell lines, the SI values were higher with NAC than with THC. These differential selectivity patterns may reflect distinct mechanisms of action and cell type-specific vulnerabilities to each compound.

Based on these results, further assays were carried out to examine how THC affects the growth of BxPC-3, Panc-1, SW480, and RKO cells.

### 3.2. Influence of Postbiotics on the Anticancer Effect of Chemotherapeutic Drugs in Pancreatic and Colorectal Cancer Cell Lines

A cell viability assay was performed to determine whether the presence of postbiotics (NAC or THC) modulated the effect of classical chemotherapeutic agents in colorectal (SW480 and RKO) and pancreatic (BxPC-3 and Panc-1) cancer cell lines, respectively, 5-FU and gemcitabine. For this analysis, the IC_50_ values of NAC and THC previously calculated (Table 1 and Table 2) were used. The concentration of 5-FU in CRC cells (46.68 µM for SW480 and 4.85 µM for RKO) was previously determined using the SRB assay [34], and the concentration of gemcitabine in PC cells (1 µM for both BxPC-3 and Panc-1) was determined using the Trypan Blue viability test [35].

#### 3.2.1. N-Acetylcysteine Enhances the Effect of Gemcitabine and 5-Fluorouracil in the Growth of BxPC-3 and RKO Cell Lines, Respectively

**Figure 3:**
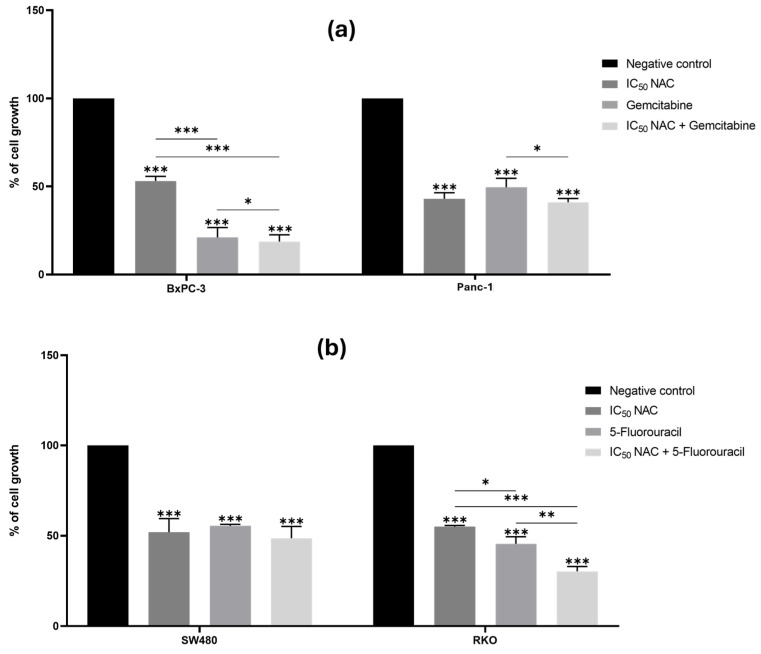
N-acetylcysteine enhances the effect of gemcitabine in BxPC-3 cells and of 5-fluorouracil in RKO cells. Bar graphs represent the mean percentage of cell growth from at least three independent experiments, with standard deviation (S.D.) shown as error bars. Columns correspond to negative control, positive control, IC_50_ NAC alone, and NAC combined with positive control. Statistical significance: *p* < 0.05 (*), *p* < 0.01 (**), and *p* < 0.001 (***). (**a**) Results for pancreatic cancer cell lines BxPC-3 and Panc-1, with gemcitabine (1 µM) as positive control. (**b**) Results for colorectal cancer cell lines SW480 and RKO, with 5-fluorouracil (46.68 µM for SW480 and 4.85 µM for RKO) as positive control. For PC cell lines, we used the Muse™ Count & Cell Viability Reagent, whilst for CRC cell lines, we performed the Sulforhodamine B assay.

Treatment of both PC and CRC cell lines with NAC at their IC_50_ values resulted in a reduction in cell viability compared to the negative control (*p* < 0.05, Figure 3). These findings support the cytotoxic effect of NAC on Panc-1, BxPC-3, SW480, and RKO cells, consistent with the results described above.

In PC cells, co-treatment with NAC and gemcitabine significantly enhanced the effect of gemcitabine in BxPC-3, but not in Panc-1 (Figure 3a). In BxPC-3 cells, cell growth was reduced to 53% with NAC, 21% with gemcitabine, and 19% with the combination, indicating an enhancement of the effect of gemcitabine. In Panc-1 cells, the combination of NAC with gemcitabine significantly reduced cell growth compared to gemcitabine alone, indicating enhanced cytotoxicity. However, no significant difference was observed between NAC treatment alone and the combination treatment, suggesting that NAC did not further enhance gemcitabine-induced growth inhibition in this cell line.

In CRC cells, NAC enhanced the effect of 5-FU in RKO, but not in SW480 (Figure 3b). In RKO, cell growth was reduced to 55% with NAC, 46% with 5-FU, and 30% with the combination.

In summary, NAC enhanced the antiproliferative effects of gemcitabine in *KRAS*^WT^ BxPC-3 cells and of 5-FU in *BRAF*-mutant RKO cells but showed limited enhancement in *KRAS*-mutant Panc-1 and SW480 cells. These differential responses suggest that genetic background and associated signaling pathways may influence the ability NAC has to potentiate the effect of chemotherapy drugs. The epithelial phenotype of BxPC-3 and certain characteristics of RKO may render these cells more susceptible to NAC-mediated chemosensitization compared to the more resistant mesenchymal-like Panc-1 and SW480 cells.

#### 3.2.2. Tetrahydro β-Carboline Carboxylic Acid Enhances the Effect of Gemcitabine on the Growth of Pancreatic Cancer Cell Lines

**Figure 4:**
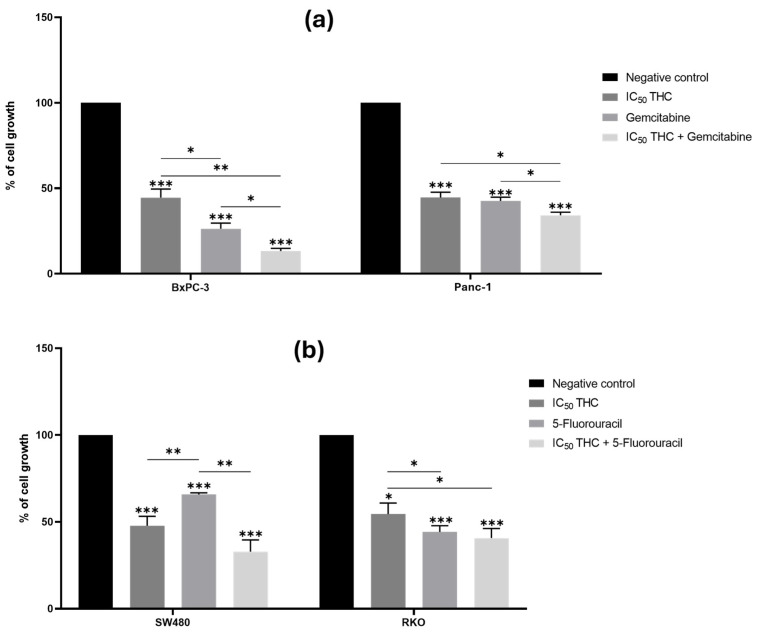
Tetrahydro β-carboline carboxylic acid enhances the effect of gemcitabine in BxPC-3 cells and Panc-1 cells but not the effect of 5-fluorouracil in colorectal cancer cell lines. Bar graphs represent the mean percentage of cell growth from at least three independent experiments, with standard deviation (S.D.) shown as error bars. Columns correspond to negative control, positive control, IC_50_ THC, and THC combined with positive control. Statistical significance: *p* < 0.05 (*), *p* < 0.01 (**), and *p* < 0.001 (***). (**a**) Results for pancreatic cancer cell lines BxPC-3 and Panc-1, with gemcitabine (1 µM) as positive control. (**b**) Results for colorectal cancer cell lines SW480 and RKO, with 5-fluorouracil (46.68 µM for SW480 and 4.85 µM for RKO) as positive control. For PC cell lines, we used the Muse™ Count & Cell Viability Reagent, whilst for CRC cell lines, we performed the Sulforhodamine B assay.

As shown in Figure 4, treatment with the IC_50_ values of THC resulted in a significant reduction in viability across all four cancer cell lines compared to the negative control.

In PC cells, THC significantly enhanced the effect of gemcitabine in both BxPC-3 and Panc-1 (Figure 4a). Cell growth was reduced to 45% (BxPC-3) and 45% (Panc-1) with THC alone, to 26% (BxPC-3) and 42% (Panc-1) with gemcitabine alone, and to 13% (BxPC-3) and 34% (Panc-1) with the combination, indicating a stronger inhibitory effect.

In CRC cells, however, THC did not enhance the effect of 5-FU in either SW480 or RKO (Figure 4b). No significant differences were observed between treatment with each compound alone and the combined treatment.

Overall, the consistent enhancement observed with gemcitabine in both BxPC-3 and Panc-1, despite the difference in *KRAS* status, suggests that the mechanisms of chemosensitization of THC may be independent of KRAS-driven pathways in pancreatic cancer. Moreover, the lack of viability enhancement of THC with 5-FU in CRC cells may indicate differences in drug mechanisms or tissue-specific factors that could affect THC bioactivity.

### 3.3. Analysis of the Effect of Newly Identified Postbiotics on Apoptosis in Pancreatic and Colorectal Cancer Cell Lines

The effect on programmed cell death, apoptosis, was quantitively analyzed using AV and PI staining. Apoptosis represents a key barrier that cancer cells must overcome to sustain growth and proliferation. It is characterized by specific physiological changes, including the externalization of phosphatidylserine to the outer leaflet of the plasma membrane during the early stages, followed in later stages by the loss of membrane integrity.

#### 3.3.1. N-Acetylcysteine Induces Apoptosis in Panc-1, SW480, and RKO and Enhances the Pro-Apoptotic Effect of Gemcitabine and 5-Fluorouracil

**Figure 5:**
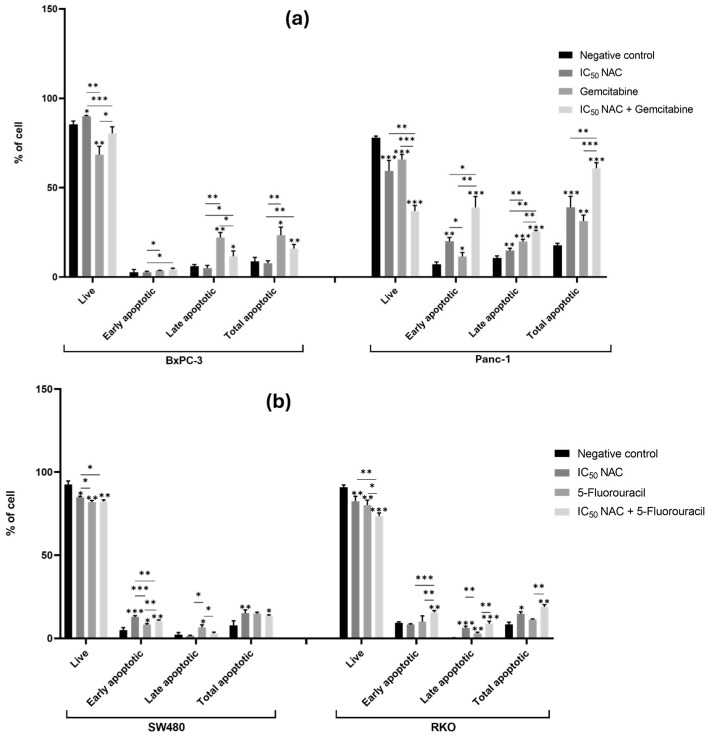
N-acetylcysteine affects apoptosis in Panc-1, SW480, and RKO cells and enhances the effect of gemcitabine and 5-fluorouracil in pancreatic and colorectal cancer cell lines, respectively. Bar graphs show the percentage of cells from at least three independent experiments, with standard deviation (S.D.) shown as error bars. The x-axis displays different cell populations for each condition (negative control, positive control, IC_50_ NAC alone, and NAC combined with positive control). Statistical significance: *p* < 0.05 (*), *p* < 0.01 (**), and *p* < 0.001 (***). (**a**) Results for pancreatic cancer cell lines BxPC-3 and Panc-1, with gemcitabine (1 µM) as positive control. (**b**) Results for colorectal cancer cell lines SW480 and RKO, with 5-fluorouracil (46.68 µM for SW480 and 4.85 µM for RKO) as positive control. Cell death was assessed by Annexin V/Propidium Iodide.

In BxPC-3 cells, NAC significantly reduced the proportion of live cells compared to the negative control but did not significantly affect the proportion of apoptotic cells. This finding suggests that NAC may induce alternative forms of cell death in BxPC-3 cells that do not involve classical apoptotic pathways, such as necroptosis, autophagy, pyroptosis, or other caspase-independent cell death mechanisms [36]. In contrast, NAC treatment in Panc-1 cells significantly decreased the percentage of live cells and increased both early and late apoptosis. Compared with the control (live 78%, early apoptotic 7%, late apoptotic 11%, total apoptotic 18%), NAC reduced live cells to 59% and increased apoptosis (early 20%, late 15%, total 39%). These findings indicate that NAC promotes apoptosis in Panc-1 cells (Figure 5a and Figure S1a).

When NAC was combined with gemcitabine, BxPC-3 cells showed significant changes in viability and apoptosis (live cells: NAC 90%, gemcitabine 69%, and combination 80%; late apoptotic cells: NAC 5%, gemcitabine 22%, and combination 12%). This suggests that NAC modifies gemcitabine’s effect by reducing its impact on both live and late apoptotic cells. In Panc-1, however, co-treatment with NAC and gemcitabine enhanced the cytotoxic effect, with reductions in live cells (NAC 59%, gemcitabine 66%, and combination 37%) and increases in early (NAC 20%, gemcitabine 12%, and combination 39%), late (NAC 15%, gemcitabine 20%, and combination 26%), and total apoptotic cells (NAC 39%, gemcitabine 31%, and combination 61%) (Figure 5a and Figure S1a). These results demonstrate that NAC enhances gemcitabine-induced apoptosis in Panc-1 cells, potentially through modulation of oxidative stress and apoptotic signaling pathways [37].

In CRC cell lines, NAC also affected apoptosis. In SW480, NAC reduced live cells from 93% (control) to 85% and increased early apoptosis from 5% to 13%, raising total apoptosis from 8% to 15%. In RKO, NAC reduced live cells (91% to 83%) and increased late apoptosis (0.3% to 6%) and total apoptosis (8% to 15%) (Figure 5b and Figure S1b).

NAC also enhanced the effect of 5-FU. In SW480, co-treatment resulted in a higher proportion of early apoptotic cells compared to NAC or 5-FU alone (NAC 13%, 5-FU 8%, and combination 11%). In RKO, co-treatment reduced live cells (NAC 83%, 5-FU 80%, and combination 74%) and increased early apoptosis (NAC 8%, 5-FU 10%, and combination 16%). These results indicate that NAC potentiates the pro-apoptotic effect of 5-FU in RKO cells (Figure 5b and Figure S1b).

In conclusion, NAC induced apoptosis in Panc-1, SW480, and RKO cells, but not in BxPC-3 cells where alternative death mechanisms may be involved. The combination of NAC with chemotherapy drugs enhanced apoptotic responses in Panc-1 and RKO cells, correlating with the viability data and suggesting that the chemosensitizing effects of NAC occur at least partially through the promotion of apoptotic cell death pathways. The cell line-specific differences in apoptotic responses align with their distinct genetic background and phenotypic characteristics.

#### 3.3.2. Tetrahydro β-Carboline Carboxylic Acid Affects and Enhances the Effect That Gemcitabine and 5-Fluorouracil Have in the Apoptosis of BxPC-3 and RKO Cells, Respectively

**Figure 6:**
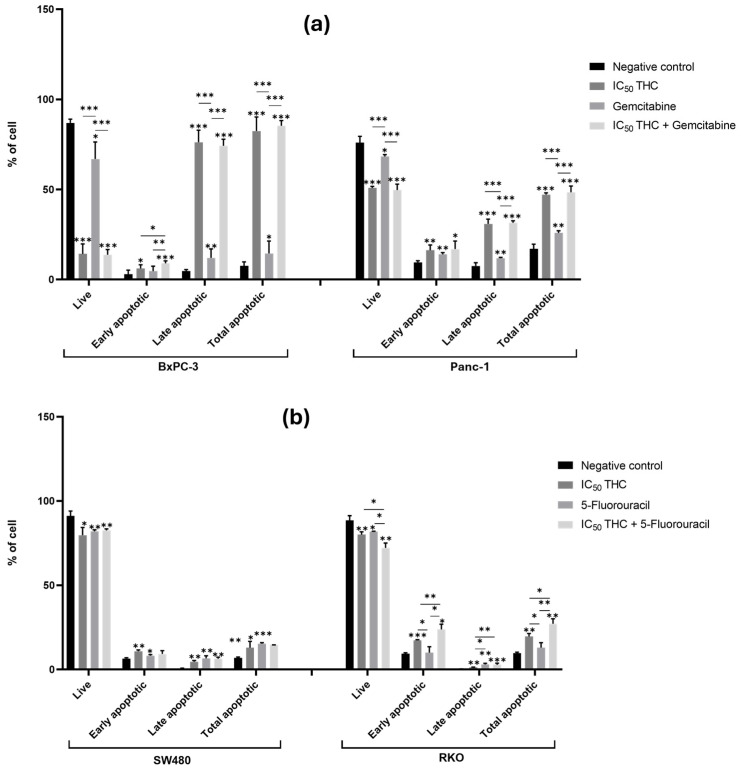
Tetrahydro β-carboline carboxylic acid affects apoptosis in pancreatic and colorectal cancer cell lines and enhances the effect of gemcitabine and 5-fluorouracil in BxPC-3 and RKO cells, respectively. Bar graphs show the percentage of cells from at least three independent experiments, with standard deviation (S.D.) shown as error bars. The x-axis displays different cell populations for each condition (negative control, positive control, IC_50_ THC, and THC combined with positive control). Statistical significance: *p* < 0.05 (*), *p* < 0.01 (**), and *p* < 0.001 (***). (**a**) Results for pancreatic cancer cell lines BxPC-3 and Panc-1, with gemcitabine (1 µM) as positive control. (**b**) Results for colorectal cancer cell lines SW480 and RKO, with 5-fluorouracil (46.68 µM for SW480 and 4.85 µM for RKO) as positive control. Cell death was assessed by Annexin V/Propidium Iodide.

THC significantly altered apoptosis in all four cancer cell lines compared to the negative control (Figure 6). In BxPC-3, THC reduced live cells from 87% to 14% and increased apoptosis from 8% to 83% (early 6% and late 76%). In Panc-1, THC reduced live cells from 76% to 51% and increased total apoptosis from 17% to 47% (early 16% and late 31%) (Figure 6a and Figure S2a). These pro-apoptotic effects demonstrate the ability THC has to trigger programmed cell death across both PC cell lines.

In BxPC-3 cells, the combination of THC and gemcitabine further increased early apoptosis compared with either treatment alone (gemcitabine 5%, THC 6%, and combination 9%), indicating that THC enhances gemcitabine’s pro-apoptotic effect. This effect, however, was not observed in Panc-1% (Figure 6a and Figure S2a).

In CRC cells, THC also significantly increased apoptosis. In SW480, THC reduced live cells (91% to 80%) and increased early apoptosis (7% to 11%), late apoptosis (0.7% to 5%), and total apoptosis (7% to 13%). In RKO, THC reduced live cells (89% to 80%) and increased early apoptosis (9% to 17%) and total apoptosis (8% to 20%) (Figure 6b and Figure S2b).

Although THC did not enhance the effect of 5-FU on viability in SW480, it did enhance apoptosis in RKO. In RKO cells, co-treatment with THC and 5-FU reduced live cells (THC 80%, 5-FU 82%, and combination 72%) and increased early apoptosis (THC 17%, 5-FU 10%, and combination 24%) and total apoptosis (THC 20%, 5-FU 13%, and combination 27%). These findings demonstrate that THC significantly enhances the pro-apoptotic effect of 5-FU in RKO cells (Figure 6b and Figure S2b).

In brief, THC induced apoptosis across all tested cell lines, with striking effects in BxPC-3 that had 83% of apoptotic cells. THC enhanced chemotherapy drug-induced apoptosis in BxPC-3 and RKO. The consistent pro-apoptotic activity of THC across diverse cells harboring distinct genetic backgrounds suggests that its mechanism of action may target pathways common to multiple cancer genotypes rather than being mutation-specific driven.

### 3.4. Analysis of the Effect of Postbiotics on the Cell Cycle of Pancreatic and Colorectal Cancer Cell Lines

Cell cycle analysis was performed by flow cytometry. Ribonuclease A (RNAse A) was added to enhance the specificity of DNA staining performed by PI, which binds stoichiometrically to nucleic acids, thereby allowing discrimination of cells of different stages of the cell cycle (G0/G1, S, and G2/M) based on DNA content.

#### 3.4.1. N-Acetylcysteine Affects the Cell Cycle of Panc-1 and Enhances the Effect of 5-Fluorouracil in Colorectal Cancer Cell Lines

**Figure 7:**
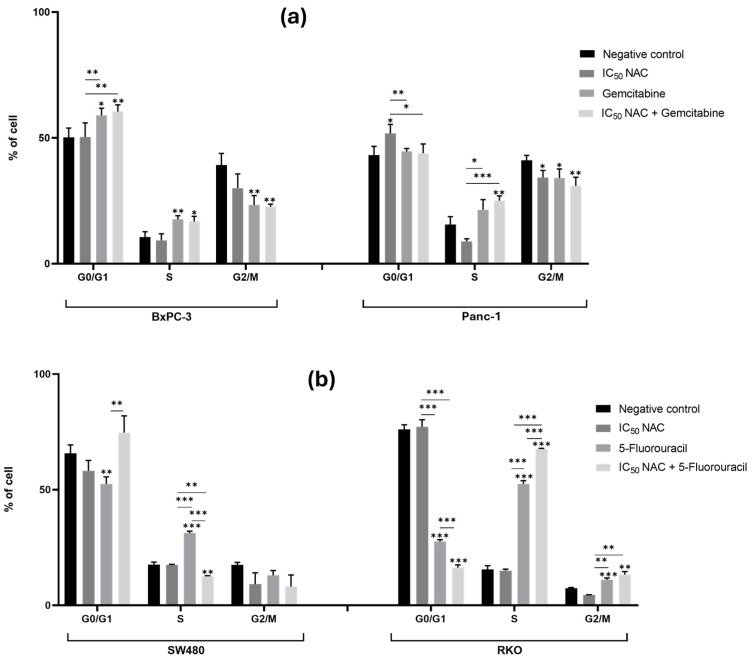
N-acetylcysteine affects the cell cycle of Panc-1 cells and enhances the effect of 5-fluorouracil in colorectal cancer cell lines. Bar graphs represent the percentage of cells from at least three independent experiments, with standard deviation (S.D.) shown as error bars. The x-axis indicates cell cycle phases for each condition (negative control, positive control, IC_50_ NAC alone, and NAC combined with positive control). Statistical significance: *p* < 0.05 (*), *p* < 0.01 (**), and *p* < 0.001 (***). (**a**) Results for pancreatic cancer cell lines BxPC-3 and Panc-1, with gemcitabine (1 µM) as positive control. (**b**) Results for colorectal cancer cell lines SW480 and RKO, with 5-fluorouracil (46.68 µM for SW480 and 4.85 µM for RKO) as positive control. Cell cycle analysis was performed by flow cytometry.

In the case of PC cell lines, Figure 7a shows that treatment with NAC did not affect the cell cycle distribution of BxPC-3 cells when compared with the negative control. Meanwhile, in Panc-1 cells, treatment with NAC resulted in a significant increase in the proportion of cells in the G0/G1 phase and a significant decrease in the proportion of cells in the G2/M phase (G0/G1: 52%; G2/M: 34%) compared with the negative control (G0/G1: 43%; G2/M: 41%). This suggests that NAC may induce G0/G1 cell cycle arrest in Panc-1 cells, potentially contributing to its antiproliferative effects. However, treatment with gemcitabine alone or in combination with NAC did not have any significant differences in cell cycle distribution in either BxPC-3 or Panc-1 cells (Figure 7a and Figure S3a).

For CRC cell lines, Figure 7b shows that NAC treatment alone did not significantly affect the cell cycle distribution of SW480 or RKO cells compared with the negative control. However, NAC enhanced the effect of 5-FU on both CRC cell lines. In SW480 cells, there was a significant difference in the proportion of cells in the S phase between treatments with NAC, 5-FU, and the combination of NAC with 5-FU (NAC: 18%; 5-FU: 31%; NAC + 5-FU: 13%). The reduced S phase in the NAC + 5-FU combination compared to 5-FU alone suggests that NAC may modulate the 5-FU mechanism of S phase arrest. In RKO cells, significant differences were observed in the G0/G1 and S phases when comparing cells treated with NAC (G0/G1: 77%; S: 15%), 5-FU alone (G0/G1: 28%; S: 53%), and the combination of NAC with 5-FU (G0/G1: 16%; S: 68%). Interestingly, the combination promoted progression into S phase compared to either NAC or 5-FU treatment alone; meanwhile, we previously observed an enhancement in apoptosis. These results could reflect complex cell cycle dynamics where cells enter S phase but fail to complete DNA synthesis due to 5-FU-mediated thymidylate synthase inhibition, which triggers apoptotic pathways [10]. The increased S-phase accumulation combined with elevated apoptosis suggests replication stress and DNA damage responses. These results suggest that NAC enhances the activity of 5-FU in regulating the cell cycle of CRC cells (Figure 7b and Figure S3b).

In summary, NAC induced G0/G1 arrest in Panc-1 cells and modulated 5-FU-mediated cell cycle effects in CRC cells, particularly promoting S-phase accumulation in RKO when combined with 5-FU. The cell cycle effects varied across cell lines and were more pronounced in combination with chemotherapy than with NAC alone, suggesting that NAC depends on cellular context and drug interactions to modulate cell cycle activity.

#### 3.4.2. Tetrahydro β-Carboline Carboxylic Acid Influences the Cell Cycle of Both Pancreatic and Colorectal Cancer Cell Lines and Enhances the Effects of Gemcitabine and 5-Fluorouracil

**Figure 8:**
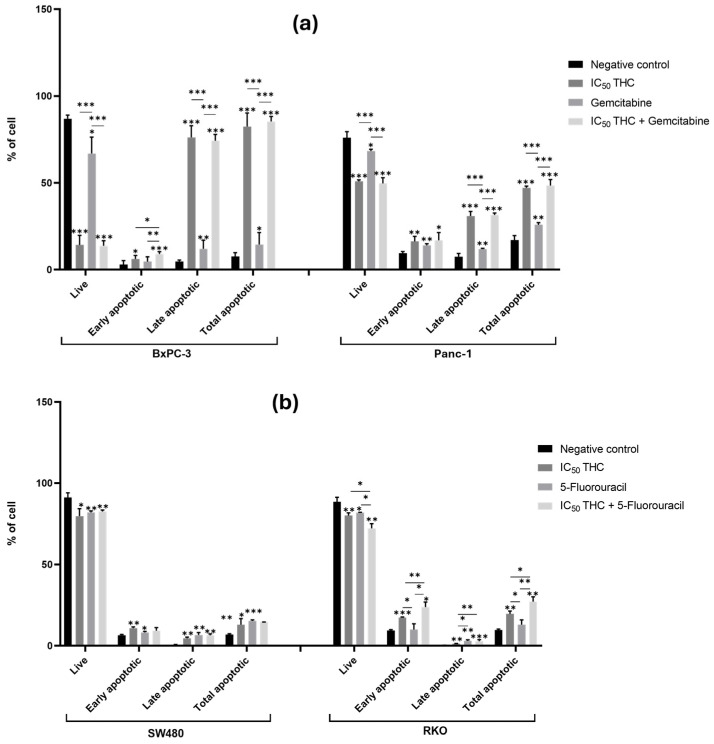
Tetrahydro β-carboline carboxylic acid affects the cell cycle of pancreatic and colorectal cancer cell lines and enhances the effect of gemcitabine and 5-fluorouracil in pancreatic cancer and SW480 cells, respectively. Bar graphs show the percentage of cells from at least three independent experiments, with standard deviation (S.D.) shown as error bars. The x-axis indicates cell cycle phases for each condition (negative control, positive control, IC_50_ THC, and THC combined with positive control). Statistical significance: *p* < 0.05 (*), *p* < 0.01 (**), and *p* < 0.001 (***). (**a**) Results for pancreatic cancer cell lines BxPC-3 and Panc-1, with gemcitabine (1 µM) as positive control. (**b**) Results for colorectal cancer cell lines SW480 and RKO, with 5-fluorouracil (46.68 µM for SW480 and 4.85 µM for RKO) as positive control. Cell cycle analysis was performed by flow cytometry.

Figure 8a illustrates the effects of THC on PC cell lines. In BxPC-3 cells, THC treatment resulted in a significant increase in the proportion of cells in the G0/G1 and G2/M phases, accompanied by a significant decrease in the S-phase population (G0/G1: 64%; S: 6%; G2/M: 31%) compared with the negative control (G0/G1: 44%; S: 24%, G2/M: 27%). S-phase depletion suggests that THC disrupts DNA replication, potentially through DNA damage induction or replication stress. In Panc-1 cells, THC induced a significant decrease in S-phase cells and a significant increase in G2/M cells (S: 9%; G2/M: 39%) compared with the negative control (S: 12%; G2/M: 23%) (Figure 8a and Figure S4a).

Furthermore, in BxPC-3 cells, a comparison of gemcitabine treatment alone with the combination of THC and gemcitabine showed a significant decrease in G0/G1-phase cells (THC: 64%; gemcitabine: 54%; THC + gemcitabine: 46%) and a significant increase in S- (THC: 6%; gemcitabine: 27%; THC + gemcitabine: 34%) and G2/M-phase cells (THC: 31%; gemcitabine: 10%; THC + gemcitabine: 18%). This suggests that the combination promotes cell cycle progression into S and G2/M phases compared to THC alone, where the nucleoside analog mechanism of gemcitabine may drive cells into S phase even despite THC-mediated disruption, which could potentially create conditions for enhanced replication stress and apoptosis. In Panc-1 cells, the combination of THC and gemcitabine significantly increased the proportion of cells in the S phase (THC: 9%; gemcitabine: 17%; THC + gemcitabine: 21%). These results indicate that THC enhances the effects of gemcitabine on the cell cycle of both BxPC-3 and Panc-1 cells (Figure 8a and Figure S4a).

In CRC cell lines, Figure 8b shows that THC significantly altered the cell cycle of SW480 cells, with differences observed in G0/G1 (68%), S (20%), and G2/M (5%) phases compared with the negative control (G0/G1: 80%; S: 11%; G2/M: 10%). In RKO cells, THC treatment significantly decreased G0/G1-phase cells (56%) and increased S-phase cells (18%) compared with the negative control (G0/G1: 76%; S: 22%) (Figure 8b and Figure S4b).

Moreover, THC enhanced the effect of 5-FU on cell cycle distribution in SW480 cells. Specifically, combined treatment with THC and 5-FU significantly reduced the proportion of cells in G0/G1 and increased the proportion of cells in the S and G2/M phases (G0/G1: 44%; S: 25%; G2/M: 19%) compared to either treatment with THC (G0/G1: 68%; S: 20%; G2/M: 5%) or 5-FU (G0/G1: 54%; S: 31%; G2/M: 12%). This enhanced S and G2/M accumulation in combination treatment suggests a disruption of cell cycle checkpoints, which may contribute to the apoptotic responses previously observed. In contrast, in RKO cells, THC did not enhance the effects of 5-FU on cell cycle distribution (Figure 8b and Figure S4b).

Summing up, THC disrupted cell cycle distribution across all cell lines, typically reducing S-phase and increasing G0/G1 and/or G2/M populations. In the case of all PC cell lines and in SW480, when combined with chemotherapy drugs, THC modulated drug-induced cell cycle changes, generally enhancing S-phase accumulation, which could contribute to replication stress and apoptosis. The consistent cell cycle disruptive effects across different genetic backgrounds support THC as a broad-spectrum cell cycle modulator, though the specific phase distribution varied by cell line and treatment combination.

## 4. Discussion

Restoring a favorable microbiota has been shown to decrease cancer progression, enhance the efficacy of chemotherapy, and reduce the toxic side effects of anticancer drugs. Consequently, the study of postbiotics in cancer-derived cell lines has gained increasing relevance. The present work aimed to evaluate the effects of two postbiotics, NAC and THC, on cell viability, cell cycle progression, and apoptosis in PC and CRC cell lines. Moreover, the study assessed whether co-administration of these postbiotics with standard chemotherapeutic agents, gemcitabine for PC and 5-FU for CRC, could potentiate their anticancer effects.

Our findings add to the emerging understanding of the microbiota–cancer–chemotherapy axis, which recognizes that gut microbiota influences cancer progression through multiple mechanisms, such as the modulation of the systemic and local immunity system, the production of metabolites that affect cancer cell signaling, the metabolism of xenobiotics, including chemotherapy drugs, and the maintenance of intestinal barrier integrity. Moreover, chemotherapy substantially alters microbiota composition, often inducing dysbiosis, which can limit treatment efficacy and increase toxicity [17,18]. This bidirectional interaction has stimulated interest in microbiota-targeted interventions to optimize cancer therapy. Our study demonstrates that specific microbiota-derived postbiotics can directly modulate cancer cell behavior and chemotherapy responses, providing molecular-level insights into potential mechanisms underlying probiotic-mediated anticancer effects. Previous work in PDAC xenograft models showed that gemcitabine induced dysbiosis with increased pro-inflammatory bacteria, while probiotic administration (PRO2101) restored beneficial microbiota and enhanced therapeutic outcomes [18,19]. Our identification of NAC and THC as bioactive metabolites from the most effective probiotic strains provides mechanistic support for how probiotics may mediate anticancer effects through production of specific bioactive metabolites.

Cancer-derived cell lines were selected for this work because they represent reliable and widely used models in cancer research, particularly for testing novel anticancer agents and therapies [38]. In PC, gemcitabine has demonstrated greater improvements in overall survival and patient-reported outcomes, such as pain reduction, compared to 5-FU, and is therefore considered the standard-of-care treatment [10,12,39,40]. Nevertheless, resistance to both 5-FU and gemcitabine frequently develop through multifactorial mechanisms. In CRC, 5-FU remains the standard adjuvant chemotherapy for early stage disease and the first-line option for metastatic CRC. The concentrations of chemotherapeutic agents used in the combination studies were based on previously published in vitro studies performed in our laboratories. For gemcitabine, a concentration of 1 μM was used for both BxPC-3 and Panc-1 cells based on Trypan Blue viability assays [35] and for 5-FU, concentrations of 46.68 μM for SW480 and 4.85 μM for RKO were previously determined using SRB assays in our laboratory [34]. While these concentrations are within the range of clinically achievable plasma concentrations for gemcitabine (1–20 μM) and 5-FU (1–10 μM in continuous infusion), in vitro studies typically use higher concentrations due to the absence of pharmacokinetic factors present in vivo. These concentrations were selected to achieve measurable cytotoxic effects while allowing the detection of potential enhancement by postbiotics.

The genetic background of the selected PC and CRC cell lines was also taken into consideration. *KRAS* is the most frequently mutated oncogene in cancer and is constitutively active in approximately 95% of PC cases. For this reason, a *KRAS*^WT^ cell line (BxPC-3) and a *KRAS*^G12D^ mutant cell line (Panc-1) were included in this study. In CRC, KRAS mutations are present in ~40% of patients, while *BRAF* mutations occur in 8–15% of cases. Importantly, *KRAS* and *BRAF* mutations are mutually exclusive [7,22,23]. Oncogenic *KRAS* mutations activate downstream signaling pathways, most notably, phosphatidylinositol 3-kinase (PI3K) and mitogen-activated protein kinase (MAPK), which regulate cell proliferation, differentiation, and survival [24,25,26,27,28,29,30,31]. Thus, to include these molecular features, two CRC cell lines were used, SW480, carrying the *KRAS*^G12V^ mutation, and RKO, harboring the *BRAF*^V600E^ mutation.

The experiments demonstrated that NAC exerts a dose-dependent cytotoxic response across the four cancer cell lines. In BxPC-3 cells, the IC_50_ value was 14.41 mM, with an SI of 5.56 when compared to the non-malignant BJ-5ta control. NAC significantly reduced BxPC-3 viability without inducing apoptosis or affecting cell cycle distribution, which suggests that NAC may induce non-apoptotic cell death pathways, such as necroptosis, autophagy, ferroptosis, or other caspase-independent mechanisms [41]. While we did not directly assess these alternative death pathways in our study, this is consistent with previous published data from [36], who demonstrated that NAC treatment of BxPC-3 cells increased both necroptosis and autophagy markers while maintaining similar apoptotic levels. Their study showed that the antioxidant activity of NAC promoted non-apoptotic death pathways through modulation of ROS-dependent signaling [36]. Future studies using specific death pathway markers, such as the LC3-II/I ratio for autophagy, MLKL phosphorylation for necroptosis, lipid peroxidation assays for ferroptosis, and pharmacological inhibitors, could help characterize the mechanisms underlying the observed cytotoxicity of NAC in BxPC-3 cells.

In Panc-1 cells, NAC displayed an IC_50_ of 32.04 mM (SI = 2.50) and induced cell cycles alterations, including an increase in G0/G1- and a decrease in G2/M-phase cells, consistent with cell cycle arrest. This finding corroborates previous reports showing NAC-mediated G0/G1 accumulation in other pancreatic cell lines [42]. Apoptosis assays further demonstrated that NAC enhances apoptotic pathways, consistent with studies in cardiac H9c2 cells and gastrointestinal cancer cell lines, where NAC activated caspase cascades, cytochrome c release, and altered Bax/Bcl-2 ratios [43,44,45]. A study in human leukemia HL-60 and U937 cells treated with NAC showed an increase in ROS levels, particularly superoxide radical O^2•−^, which is converted into H_2_O_2_ by superoxide dismutase (SOD), ultimately leading to cell death, which could indicate an increase in cell damage and death [46].

In SW480 cells, the IC_50_ was 29.34 mM (SI = 1.80). Although NAC did not significantly alter the cell cycle, it reduced viability by increasing apoptotic cell populations. However, contradictory evidence exists; for instance, NAC has been shown to reduce ROS and apoptosis in other CRC lines exposed to pro-apoptotic agents [46]. In RKO cells, the IC_50_ was 22.13 mM (SI = 2.39). NAC did not significantly affect the cell cycle but decreased live cell populations while increasing apoptotic cells, suggesting activation of apoptotic pathways. Interestingly, NAC has also been reported to enhance necrosis and inflammatory responses through TNF-α/TNF-R signaling, which could influence the tumor microenvironment by promoting immune activity but also potentially harming surrounding tissues [20,47,48].

Overall, the mechanism of action of NAC appears to be context-dependent since it varies with line characteristics, basal oxidative status, and genetic background. The differences in the responses observed between BxPC-3 and Panc-1 highlight the importance of KRAS status and EMT phenotype since BxPC-3 may have different redox homeostasis and stress response pathways compared to Panc-1, which is due to the KRAS mutation in Panc-1 that activates constitutive PI3K/AKT and MAPK signaling, which can alter how cells respond to NAC-induced oxidative stress. Additionally, the mesenchymal phenotype associated with the EMT of Panc-1 can confer both enhanced migration capability and altered sensitivity to oxidative stress-based therapies [49,50]. In CRC cell lines versus normal colon NCM460 cells, the higher IC_50_ of CRC cells and the SI > 1 suggest that cancer cells may be more vulnerable to NAC-induced oxidative stress due to their elevated basal ROS levels [51].

THC exhibited greater potency than NAC, with lower IC_50_ values across all cancer cell lines. In BxPC-3 cells, THC showed an IC_50_ of 6.05 mM (SI = 1.86) and induced cell cycle arrest, reducing S-phase cells while increasing G0/G1 and G2/M populations. This suggests disrupted DNA replication and potential apoptosis, confirmed by increased apoptotic cell populations. Similar effects were observed in Panc-1 cells (IC_50_ = 7.47 mM; SI = 1.51), where THC reduced S-phase cells and increased G2/M populations, consistent with apoptosis induction.

THC was particularly effective in CRC cell lines. In SW480, it displayed an IC_50_ of 2.22 mM (SI = 9.29), inducing apoptosis by promoting S-phase accumulation and reducing G0/G1 and G2/M phases. In RKO cells, THC (IC_50_ =2.90 mM; SI = 7.11) disrupted cell cycle regulation, decreasing G0/G1 and S phases while increasing apoptotic populations. Based on our cell cycle data showing S phase reduction and G2/M accumulation, combined with robust increases in apoptotic populations, THC appears to induce cell cycle disruption followed by apoptotic cell death. Published studies on β-carboline derivatives provide insights into potential molecular mechanisms underlying the effect of THC. One study demonstrated that THC induced apoptosis in HCT-8 colorectal cancer cells through an increased Bax/Bcl-2 ratio, cytochrome c release from mitochondria, caspase 3/8/9 activation, and suppression of NF-κB signaling via the inhibition of IκB-α phosphorylation [21]. Another study showed that β-carboline-salicylic acid hybrids caused mitochondrial depolarization, increased Bax/Bcl-2 ratios, and activated caspase cascades in multiple cancer types, including colon, liver, and lung cancers [52]. Additionally, molecular modeling studies indicate that tetrahydro-β-carboline derivatives specifically bind to the catalytic site of phosphodiesterase-5 (PDE5) [53], and PDE5 inhibition has been linked to enhanced chemotherapy sensitivity in various cancers since it is overexpressed in colorectal, pancreatic, lung, and bladder cancers [54,55]. Future mechanistic studies should directly assess mitochondrial membrane potential, caspase activity, DNA damage using markers, and specific signaling pathway components, to elucidate the mechanism of action THC has in pancreatic and colorectal cancer cells.

For both postbiotics, SI values were consistently >1, indicating that higher concentrations were required to inhibit normal cells compared to cancer cells, suggesting a higher selectivity and sensitivity of cancer cells to NAC and THC and a reduced cytotoxicity toward non-malignant control cells in vitro [56]. Nevertheless, the observed SI values provide only preliminary evidence of a therapeutic reality, since in vitro selectivity indices do not always translate to in vivo selectivity.

The calculated IC_50_ values for both PC and CRC cell lines are in accordance with previous doses used in another work where the IC_50_ dose calculated for bladder cancer cells was 33.33 mM [57]. In [21] it was observed that THC had a dose-dependent response at 0.5–4 μM in HCT-8 cells with an IC_50_ of 4 μM, which is almost 1000× lower than the observed IC_50_ values in the PC and CRC cell lines in our study [21]. The difference in the sensitivity of SW480 and RKO cells when compared to HCT-8 cells could be explained by the mutations present in SW480 and RKO, while the difference between the PC cell lines and the HCT-8 cells suggests that the colorectal HCT-8 cell line is more sensitive to PC cells, which is corroborated by an in vitro study performed to understand the sensitivity of colorectal and pancreatic cancer to 5-FU in 2D and 3D culture models, which showed that colorectal SW948 and HCT116 cells were more sensitive than pancreatic MIA PaCa-2 and Panc-1 cells [58]. Notably, Panc-1 cells exhibited higher IC_50_ values than BxPC-3 cells, potentially due to their EMT phenotype, which is associated with increased invasiveness and drug resistance, since the acquisition of an EMT phenotype leads to a loss of cell–cell adhesion due to decreased expression of proteins involved in cell junctions, such as E-cadherin and claudins, and increased invasion ability through acquired cell motility [49,50]. Similarly, RKO cells showed lower IC_50_ values than SW480, consistent with EMT features and enhanced invasiveness [59]. These differences highlight how genetic background and phenotypic characteristics can influence postbiotic sensitivity. Future studies could evaluate the effects of NAC and THC on cancer cell migration and invasion, given that previous work demonstrated that *Bifidobacterium* cell-free supernatants prevented epithelial–mesenchymal transition (EMT) in BxPC-3 cells [19]. Such investigations would clarify whether these postbiotics exert antimetastatic effects beyond their antiproliferative and pro-apoptotic activities. Given that EMT is associated with metastatic potential and therapy resistance, the assessment of EMT markers alongside functional assays would be particularly valuable.

The IC_50_ values for NAC (14–32 mM) and THC (2–7 mM) substantially exceed plasma concentrations typically achieved through oral supplementation in humans. Standard NAC dosing (600–1800 mg/day) results in plasma concentrations of approximately 5–20 mM [60,61,62], which is 700–6000-fold lower than our effective concentrations. Similarly, while specific pharmacokinetic data for THC are limited, β-carboline derivatives generally show limited bioavailability [63,64,65]. These high in vitro concentrations may not be directly translatable to clinical settings without optimization of delivery methods, formulation strategies, such as nanoparticle encapsulation, liposomal delivery, or local administration approaches [66,67,68]. Alternatively, these compounds might achieve relevant concentrations in the intestinal lumen or tumor microenvironment following probiotic administration, where local production by gut microbiota could generate higher concentrations than systemic supplementation [69,70]. Future studies should investigate pharmacokinetics, tissue distribution, and tumor penetration to assess clinical feasibility.

Both postbiotics seem promising and because of the known effects of postbiotics in enhancing chemotherapy drugs efficacy [18,71], combination studies were performed to assess whether NAC and THC could potentiate the effects of standard chemotherapy drugs. This study revealed cell line-specific and drug-specific patterns of enhancement that are likely to reflect the complex interplay between genetic background, signaling pathway dependencies, and drug mechanisms of action.

NAC enhanced gemcitabine efficacy in reducing viability in BxPC-3 cells and while the same was not observed for Panc-1, NAC did significantly enhance gemcitabine efficacy in apoptosis. This difference in response can be explained by the difference in *KRAS* status since the *KRAS* mutation (*KRAS*^G12D^) present in Panc-1 allows the constitutive activation of survival pathways, such as PI3K/AKT and MAPK, which can provide resistance mechanisms [26]. Moreover, the mesenchymal characteristics of Panc-1 confer enhanced antioxidant capacity and adaptative stress responses that can counteract the effect of NAC. Lastly, epithelial BxPC-3 cells may be more vulnerable to NAC-induced oxidative stress modulation than mesenchymal Panc-1 cells. NAC mechanisms of enhancement in BxPC-3 could possibly involve modulation of NF-κB activity and ROS production, thus sensitizing cells to gemcitabine, as previously reported [20,72].

Similarly, NAC enhanced 5-FU effects in RKO but not SW480 cells. This suggests that *BRAF*-mutant cells may respond differently to NAC-mediated oxidative modulation compared to *KRAS*-mutant cells. Previous studies have shown that *BRAF*^V600E^ creates distinct signaling dependencies compared to *KRAS* mutations, with differential effects on metabolic reprogramming, oxidative stress responses, and apoptotic thresholds [73,74]. Interestingly, NAC promoted progression into S phase in RKO cells when combined with 5-FU, which likely reflects complex cell cycle dynamics where cells enter S phase but fail to complete DNA synthesis due to 5-FU-mediated thymidylate synthase inhibition, which ultimately triggers apoptotic pathways through replication stress [20,46,47,48].

THC demonstrated more consistent enhancement with gemcitabine in both BxPC-3 and Panc-1 cells, regardless of *KRAS* status, which suggests that the chemosensitizing mechanisms of THC may be independent of KRAS-driven pathways in pancreatic cancer. These results suggest that the enhancement across both PC cell lines could involve a potent disruption of multiple survival pathways through mechanisms independent of *KRAS* status or it could be through a strong induction of oxidative stress that exceeds cellular antioxidant capacity regardless of the baseline oxidative status. Another alternative is that we could have potential PDE5 inhibition, which has been previously reported to enhance gemcitabine sensitivity [53,75,76,77].

However, no significant enhancement was observed with 5-FU in CRC lines, though it did enhance apoptosis in RKO. Therefore, this cancer type-specific pattern may reflect a difference in PDE5 expression levels between pancreatic and colorectal tissues or this difference could be due to the different mechanisms of interactions the chemotherapy drug has with THC. Moreover, pancreatic and colorectal cancers have distinct metabolic profiles that may affect THC bioactivity and metabolism.

Our findings also connect to growing interest in traditional medicine-derived compounds for cancer therapy. While NAC is a synthetic derivative of the amino acid cysteine, it shares functional similarities with sulfur-containing compounds found in traditional medicinal plants and foods, such as allicin from garlic, *Allium sativum*, [78,79] and sulforaphane from cruciferous vegetables, [80,81] which have been used in traditional medicine systems for their health-promoting properties. β-carboline alkaloids, including compounds structurally related to THC, are naturally present in various medicinal plants traditionally used in Chinese, Ayurvedic, and South American medicine, including *Peganum harmala*, *Passiflora* species, and *Banisteriopsis cappi* [82,83,84]. These plants have been traditionally used for various ailments, and modern research has revealed anticancer properties of their β-carboline constituents. For instance, harmine and harmaline (β-carbolines) have shown antiproliferative effects in various cancer models [85,86,87]. The connection between gut microbiota and traditional medicine is particularly intriguing, since many beneficial effects of traditional herbal medicine may be mediated through microbiota modulation and the production of bioactive metabolites [88,89]. Several studies have shown that traditional medicine-derived compounds enhance chemotherapy efficacy, similar to our postbiotic findings. For example, curcumin (from turmeric used in Ayurvedic medicine) and berberine (from various traditional medicinal plants) have been shown to sensitize pancreatic and colorectal cancer cells to chemotherapy through mechanisms overlapping with those observed (apoptosis induction, cell cycle modulation, and ROS regulation) [90,91]. This suggests that traditional medicine knowledge, when combined with modern microbiota research and metabolomic approaches, may help identify novel therapeutic strategies. Our work demonstrates that microbiota-produced metabolites can recapitulate some beneficial effects attributed to traditional medicines, suggesting a mechanistic link between dietary interventions, microbiota modulation, and cancer therapy enhancement.

## 5. Conclusions

This study demonstrated that the postbiotics NAC and THC exert anticancer effects in PC and CRC cell lines by modulating cell viability, cell cycle progression, and apoptosis. Both compounds exhibited dose-dependent cytotoxicity with selectivity indices above one, showing that higher concentrations were required to affect non-malignant cells compared to cancer cells, suggesting more selectivity and specificity towards cancer cells; however, an assessment of systemic toxicity in vivo is needed to confirm true cancer selectivity. Importantly, THC consistently disrupted the cell cycle and induced apoptosis across all tested cell lines, indicating broader and increased anticancer activity compared with NAC across all diverse genetic backgrounds.

Furthermore, the co-administration of NAC or THC with standard chemotherapeutic drugs enhanced the anticancer efficacy of these agents in specific cell lines in a genotype-dependent manner. The combination treatments reduced cell viability and promoted apoptosis more effectively than single treatments in several contexts, particularly THC with gemcitabine in PC cells and NAC with the chemotherapy drug in a specific genetic background.

Our study adds molecular-level insights into the microbiota–cancer–chemotherapy axis, demonstrating that specific microbiota-derived metabolites can directly modulate cancer cell behavior and chemotherapy responses. This supports a model where probiotic supplementation during chemotherapy could potentially restore beneficial microbiota composition disrupted by chemotherapy, deliver anticancer metabolites locally to gastrointestinal tumors, and enhance chemotherapy efficacy through metabolite-mediated sensitization. Extensive preclinical studies including in vivo efficacy, comprehensive toxicity profiling, pharmacokinetic optimization, and mechanistic validation are essential prerequisites before any clinical consideration can be undertaken.

Overall, this work provides foundational in vitro evidence that postbiotics can differentially modulate cancer cell behavior and potentially enhance chemotherapy responses depending on the genetic background of the tumor. The differential responses across cell lines with distinct *KRAS*/*BRAF* mutation status and epithelial/mesenchymal phenotypes provide insights into factors that may predict postbiotic efficacy and suggest that patient stratification based on tumor genetic and phenotypic characteristics may be important in future clinical applications of postbiotics and microbiota modulation.

## Figures and Tables

**Table 1:** IC_50_ values of N-acetylcysteine (NAC) in pancreatic cancer (PC) cell lines (BxPC-3 and Panc-1), colorectal cancer (CRC) cell lines (SW480 and RKO), and corresponding control cell lines (BJ-5ta and NCM460) after 48 h incubation. Values are presented as mean ± S.D. The selectivity index (SI) was calculated as the ratio of IC_50_ values in control versus cancer cell lines.

IC_50_ NAC (mM)		Selectivity Index (SI)
BxPC-3	14.41 ± 0.51	5.56
Panc-1	32.04 ± 1.44	2.50
BJ-5ta	80.09 ± 1.17	-
SW480	29.34 ± 3.70	1.80
RKO	22.13 ± 1.40	2.39
NCM460	52.94 ± 5.20	-

**Table 2:** IC_50_ values of tetrahydro-β-carboline carboxylic acid (THC) in pancreatic cancer (PC) cell lines (BxPC-3 and Panc-1), colorectal cancer (CRC) cell lines (SW480 and RKO), and corresponding control cell lines (BJ-5ta and NCM460) after 48 h incubation. Values are presented as mean ± S.D. The selectivity index (SI) was calculated as the ratio of IC_50_ values in control versus cancer cell lines.

IC_50_ THC (mM)		Selectivity Index (SI)
BxPC-3	6.05 ± 0.82	1.86
Panc-1	7.47 ± 1.60	1.51
BJ-5ta	11.26 ± 2.24	-
SW480	2.22 ± 0.13	9.29
RKO	2.90 ± 0.36	7.11
NCM460	20.62 ± 0.71	-

## Data Availability

The raw data supporting the conclusions of this article will be made available by the authors on request.

## Supplementary Materials

The following supporting information can be downloaded at: https://www.mdpi.com/article/10.3390/cancers18030369/s1. Figure S1. Representative flow cytometry dot plots of apoptosis analysis by Annexin V/PI showing the effects of N-acetylcysteine (NAC) in pancreatic cancer cell lines (BxPC-3 and Panc-1) (a) and colorectal cancer cell lines (SW480 and RKO) (b); Figure S2. Representative flow cytometry dot plots of apoptosis analysis by Annexin V/PI showing the effects of Tetrahydro β-carboline carboxylic acid (THC) in pancreatic cancer cell lines (BxPC-3 and Panc-1) (a) and colorectal cancer cell lines (SW480 and RKO) (b); Figure S3. Representative histograms of cell cycle phase distributions after N-acetylcysteine (NAC) exposure in pancreatic cancer cell lines (BxPC-3 and Panc-1) (a) and colorectal cancer cell lines (SW480 and RKO) (b), assessed by PI staining; Figure S4. Representative histograms of cell cycle phase distributions after Tetrahydro β-carboline carboxylic acid (THC) exposure in pancreatic cancer cell lines (BxPC-3 and Panc-1) (a) and colorectal cancer cell lines (SW480 and RKO) (b), assessed by PI staining.

## Abbreviations

The following abbreviations are used in this manuscript:

5-FU	5-Fluorouracil
AV	Annexin V
BRAF	B-Raf proto-oncogene
CFSs	Cell-free supernatants
CRC	Colorectal cancer
CDKN2A	Cyclin-dependent kinase inhibitor 2A
DMEM	Dulbecco’s Modified Eagle’s Medium
DMSO	Dimethyl sulfoxide
DNA	Deoxyribonucleic acid
EMT	Epithelial–mesenchymal transition
FBS	Fetal bovine serum
IC50	Half-maximal inhibitory concentration
KRAS	Kirsten RAS
NRAS	Neuroblastoma RAS viral oncogene homolog
MAPK	Mitogen-activated protein kinase
NAC	N-acetylcysteine
PBS	Phosphate buffer saline
PC	Pancreatic cancer
PDAC	Pancreatic ductal adenocarcinoma
PDE5	Phosphodiesterase 5
PI	Propidium iodide
PI3K	Phosphatidylinositol 3-kinase
PI3KCA	Phosphatidylinositol-4,5-bisphosphate 3-kinase catalytic subunit alpha
PS	Phosphatidylserine
RNAse A	Ribonuclease A
ROS	Reactive oxygen species
RPMI	Roswell Park Memorial Institute Medium
SD	Standard deviation
SI	Selectivity index
SMAD4	Mothers against decapentaplegic 4
SOD	Superoxide dismutase
SRB	Sulforhodamine B assay
THC	Tetrahydro β carboline carboxylic acid
TNF-R	Tumor necrosis factor receptor
TNFα	Tumor necrosis factor α
TP53	Tumor protein 53
