# Supplementary material for: Harnessing Postbiotics to Boost Chemotherapy: N-Acetylcysteine and Tetrahydro β-Carboline Carboxylic Acid as Potentiators in Pancreatic and Colorectal Cancer
Source: Cancers (Basel). 2026 Jan 25;18(3):369. doi: 10.3390/cancers18030369 (PMC12896410; doi:10.3390/cancers18030369)
Supplement: Supplementary file 1 [file cancers-18-00369-s001.zip › cancers-4081270-supplementary.pptx]

## Slide 1
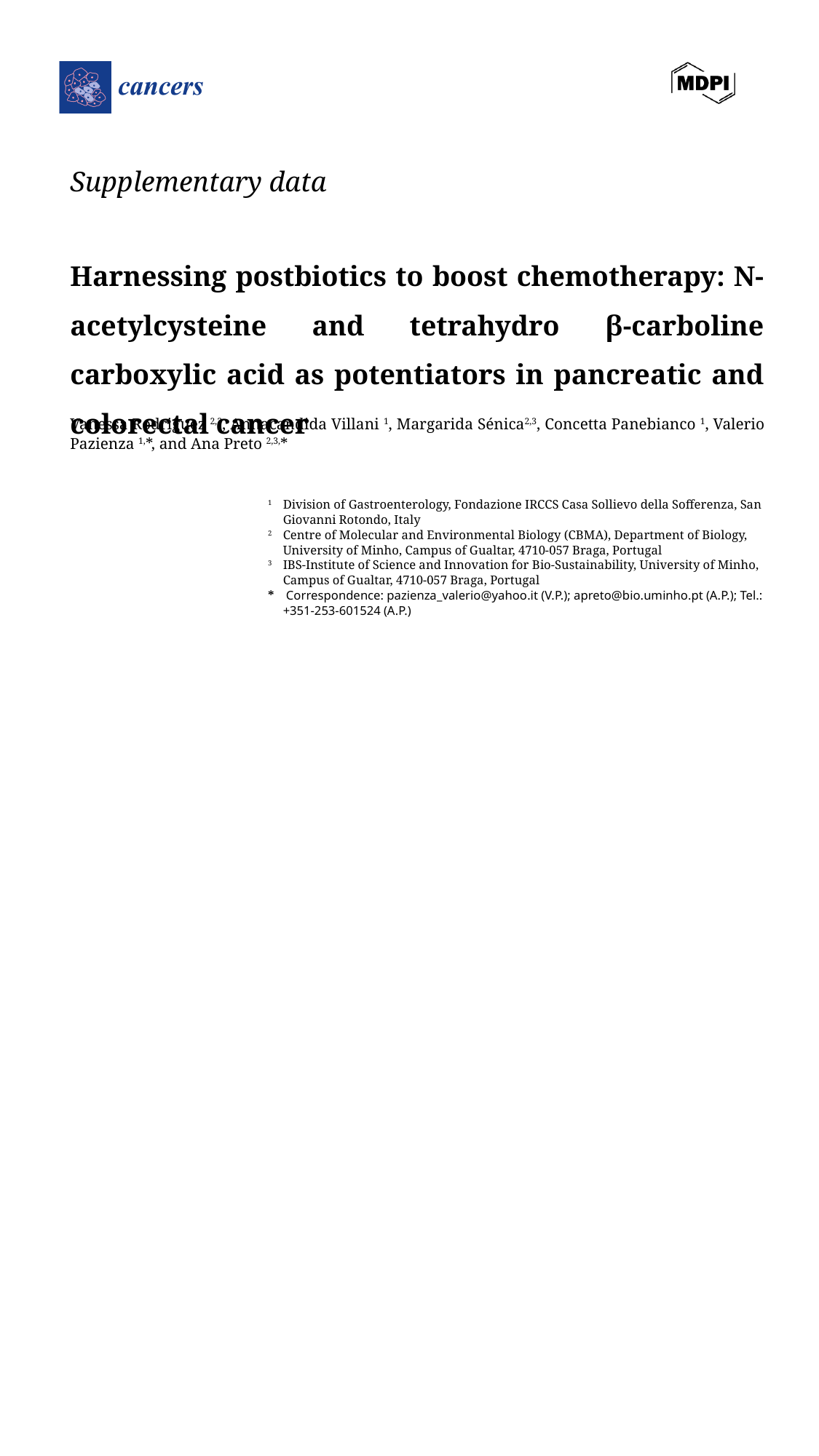

Supplementary data
Harnessing postbiotics to boost chemotherapy: N-acetylcysteine and tetrahydro β-carboline carboxylic acid as potentiators in pancreatic and colorectal cancer
Vanessa Rodriguez 2,3, Annacandida Villani 1, Margarida Sénica2,3, Concetta Panebianco 1, Valerio Pazienza 1,*, and Ana Preto 2,3,*
1	Division of Gastroenterology, Fondazione IRCCS Casa Sollievo della Sofferenza, San Giovanni Rotondo, Italy
2	Centre of Molecular and Environmental Biology (CBMA), Department of Biology, University of Minho, Campus of Gualtar, 4710-057 Braga, Portugal
3	IBS-Institute of Science and Innovation for Bio-Sustainability, University of Minho, Campus of Gualtar, 4710-057 Braga, Portugal
*	 Correspondence: pazienza_valerio@yahoo.it (V.P.); apreto@bio.uminho.pt (A.P.); Tel.: +351-253-601524 (A.P.)

## Slide 2
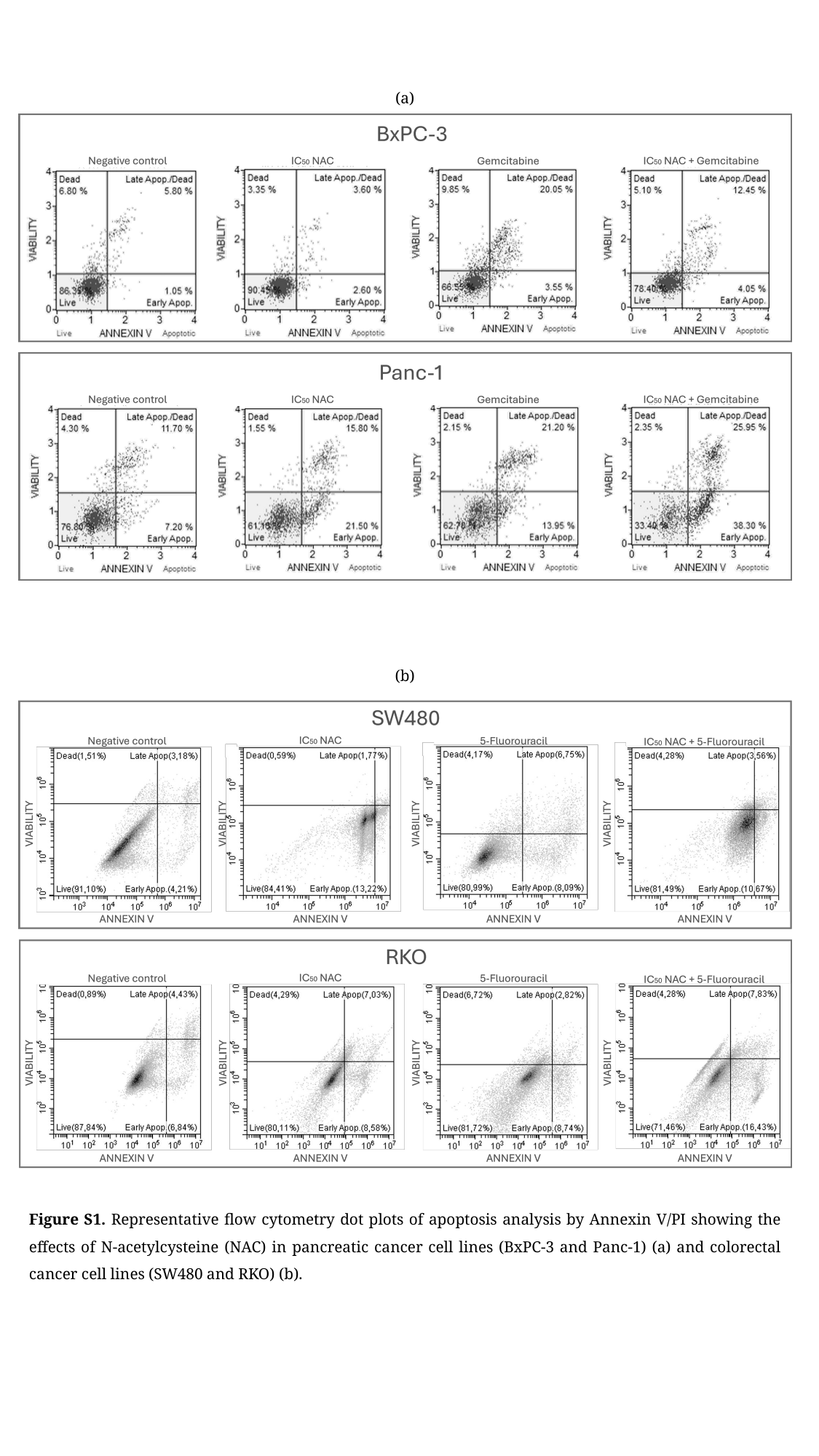

(a)
(b)
Figure S1. Representative flow cytometry dot plots of apoptosis analysis by Annexin V/PI showing the effects of N-acetylcysteine (NAC) in pancreatic cancer cell lines (BxPC-3 and Panc-1) (a) and colorectal cancer cell lines (SW480 and RKO) (b).

## Slide 3
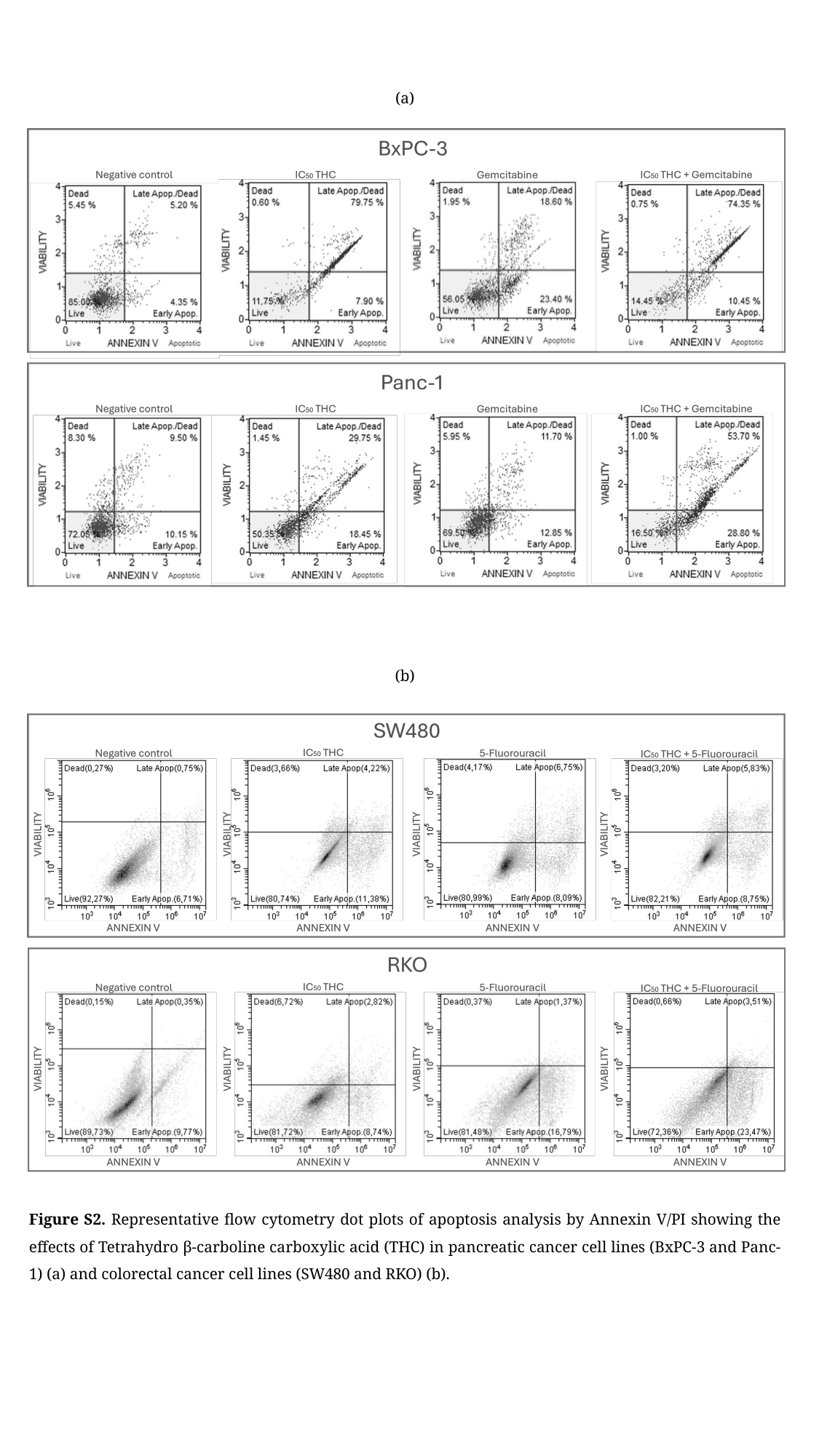

(a)
(b)
Figure S2. Representative flow cytometry dot plots of apoptosis analysis by Annexin V/PI showing the effects of Tetrahydro β-carboline carboxylic acid (THC) in pancreatic cancer cell lines (BxPC-3 and Panc-1) (a) and colorectal cancer cell lines (SW480 and RKO) (b).

## Slide 4
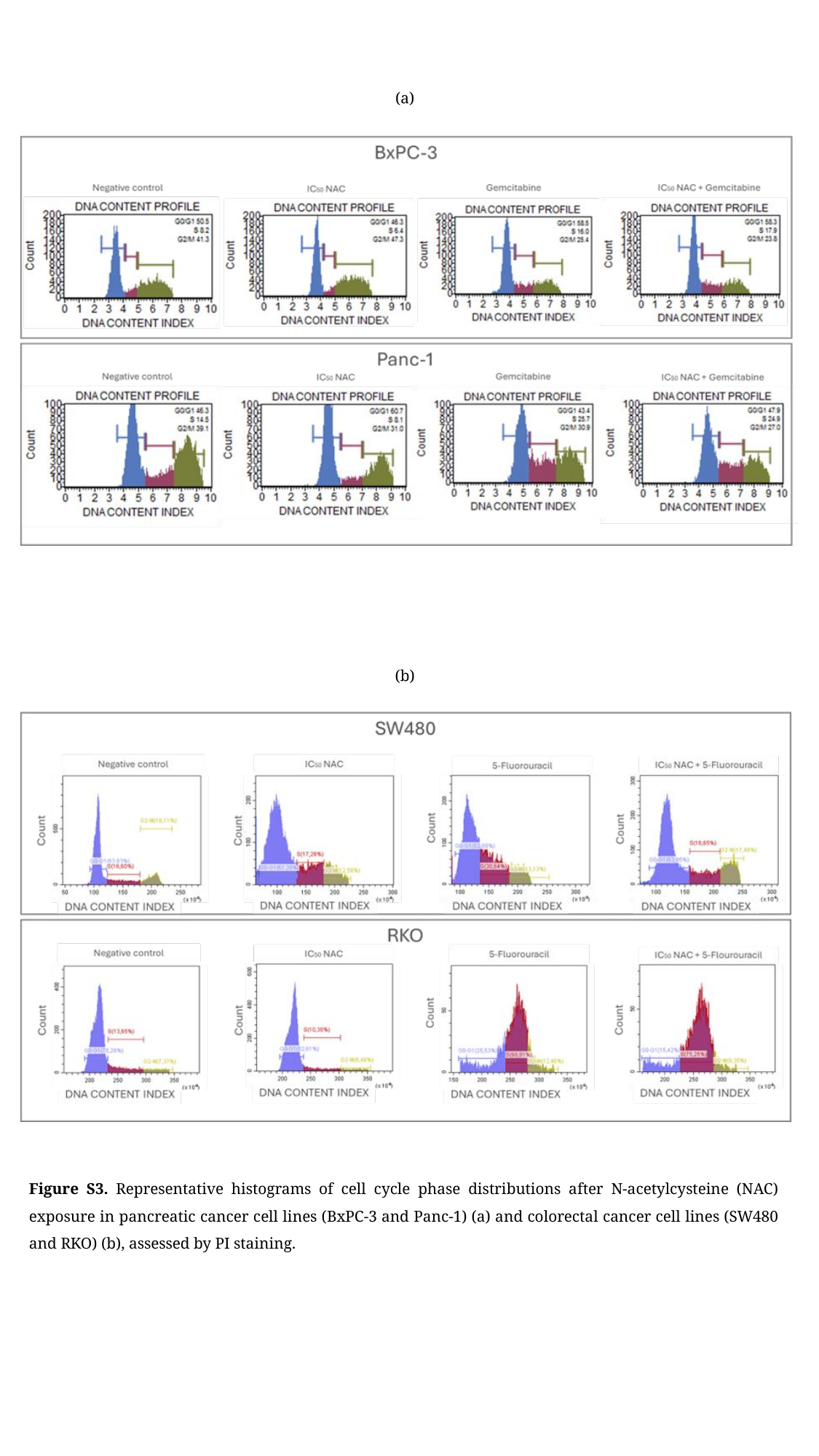

(a)
(b)
Figure S3. Representative histograms of cell cycle phase distributions after N-acetylcysteine (NAC) exposure in pancreatic cancer cell lines (BxPC-3 and Panc-1) (a) and colorectal cancer cell lines (SW480 and RKO) (b), assessed by PI staining.

## Slide 5
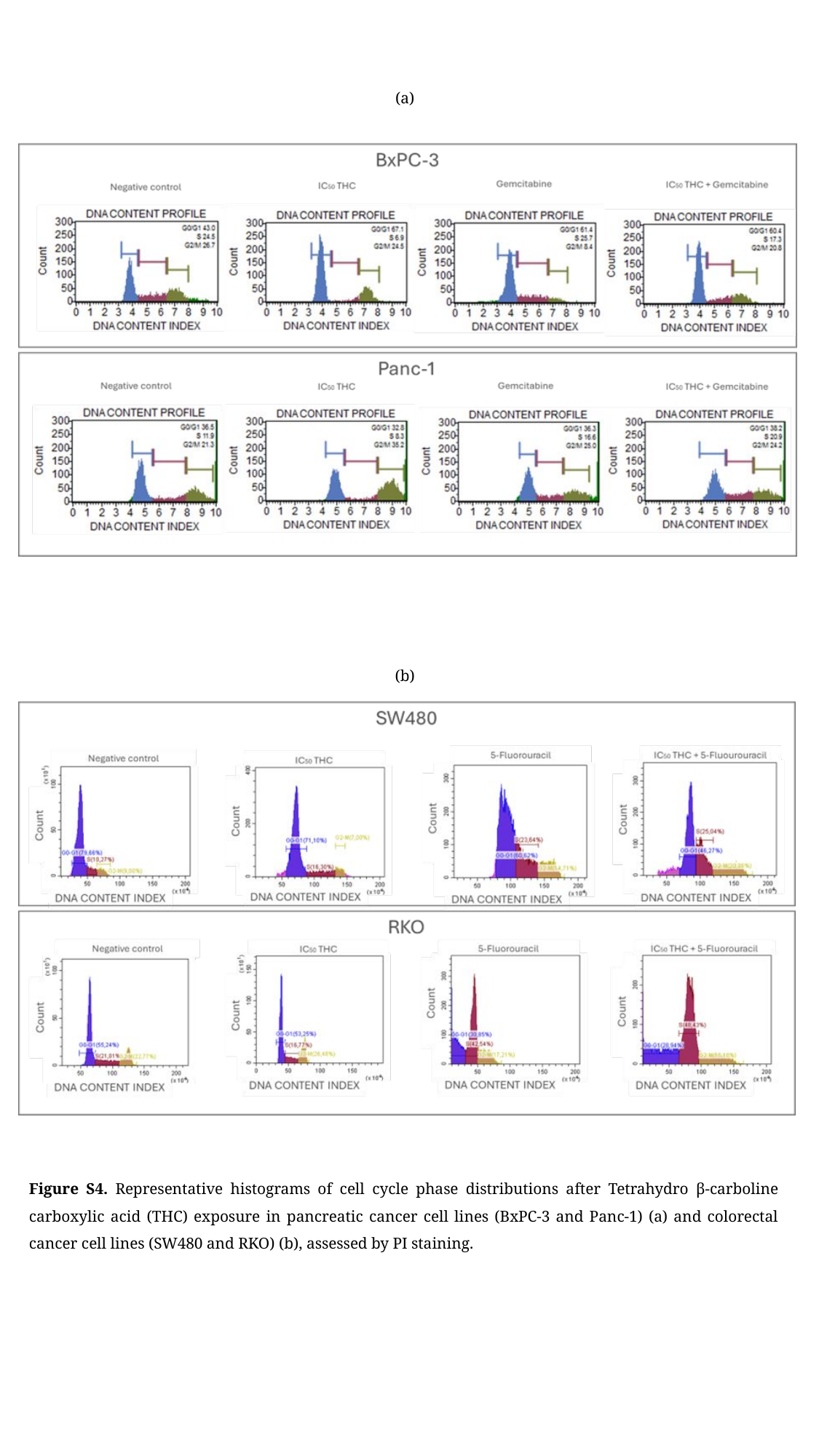

(a)
(b)
Figure S4. Representative histograms of cell cycle phase distributions after Tetrahydro β-carboline carboxylic acid (THC) exposure in pancreatic cancer cell lines (BxPC-3 and Panc-1) (a) and colorectal cancer cell lines (SW480 and RKO) (b), assessed by PI staining.
